# Diagnostic and prognostic value of FDG PET-CT in patients with suspected recurrent thymic epithelial tumors

**DOI:** 10.1038/s41598-021-00003-4

**Published:** 2021-10-15

**Authors:** Guozhu Hou, Yuanyuan Jiang, Fang Li, Wuying Cheng

**Affiliations:** 1 Department of Nuclear Medicine, State Key Laboratory of Complex Severe and Rare Diseases, Peking Union Medical College Hospital, Chinese Academy of Medical Sciences and Peking Union Medical College, Beijing, 100730 China; 2 Beijing Key Laboratory of Molecular Targeted Diagnosis and Therapy in Nuclear Medicine, Beijing, 100730 China

**Keywords:** Medical research, Oncology

## Abstract

This study aimed to evaluate the diagnostic and prognostic value of FDG PET/CT in patients with suspected recurrent thymic epithelial tumors (TETs). We retrospectively reviewed 83 patients with histopathologically proven TETs (50 thymomas; 33 thymic carcinomas) who underwent FDG PET/CT after surgery. The sensitivity and specificity of FDG PET/CT in detecting recurrence were calculated. The progression-free survival rate (PFS) was calculated by the Kaplan–Meier method. FDG PET/CT results were positive in 50 patients and negative in 33. Recurrent TETs were confirmed in 40 of 50 patients with positive PET/CT findings. The sensitivity and specificity of FDG PET/CT were 100% and 76.7%, respectively. Disease progression occurred in 28 patients during the follow-up. FDG PET/CT showed added prognostic value over the Masaoka stage and histopathology. Among patients with the same Masaoka stage, negative PET/CT was significantly associated with better PFS (*P* < 0.001). Similarly, among patients with the same histopathology, negative PET/CT was also associated with a significantly longer PFS (*P* < 0.001). FDG PET/CT demonstrated a good diagnostic performance in patients with recurrent TETs and had an important prognostic value in assessing the risk of disease progression.

## Introduction

Thymic epithelial tumors (TETs), including thymoma and thymic carcinoma, represent the most common primary neoplasms in the anterior mediastinum^1^. According to the World Health Organization (WHO) histological classification system, TETs are classified into thymomas and thymic carcinomas^2^. Thymomas are further divided into five different subtypes (A, AB, B1, B2, and B3). The prognosis of patients with thymomas is progressively worse from type A to B3^3^. Compared with thymoma, thymic carcinoma shows even more aggressive clinical behavior with a worse prognosis^4^. Another important prognostic factor for TETs is the Masaoka staging system, which is the most adopted staging system in this entity and is based on the level of invasion^5^.

Surgery is the mainstay of treatment for TETs, and complete resection is one of the most important prognostic factors. TETs are known to have a possibility of recurrence even after complete resection. In the research of the Japanese Association for research on thymus, among 2835 patients who received surgical resection of TETs, 420 (14.8%) developed recurrence^6^. In the study of the Japanese Association for chest surgery, 7.8% of patients with thymoma experienced recurrence^7^. In thymic carcinoma, 51% of patients developed recurrence^7^. Most recurrences are local and regional^8,9^, distant recurrence occurs in less than 5% of the cases^10^. Early detection of recurrence is necessary, because further treatment contributed to higher survival rates^6^.

FDG PET/CT has become an important tool for the diagnosis, staging, and re-staging of malignant tumors. FDG PET/CT is widely used in preoperative evaluation of TETs, and there is a good correlation between tumor metabolic activity and histopathological classification^11–17^. Its performance in detecting recurrent lesions and the ability to evaluate curative effects have also been reported in the literature^18–20^. Still, its impact on prognosis during the restaging process remains to be elucidated. Therefore, the aims of this study were: (1) to investigate the diagnostic performance of FDG PET/CT for suspected recurrent TETs, (2) to evaluate the added prognostic role of FDG PET/CT associated with other clinical parameters (histopathology and Masaoka stage) in the restaging process.

## Results

### Patients

A total of 83 patients (41 men, 42 women) met the inclusion criteria and were included in this study. The clinical characteristics of patients were summarized in Table 1. The median age was 52 years (range, 19‒83; mean 52.2 ± 12.9). All patients underwent surgery to remove the primary thymic tumor. Sixty-two patients also received adjuvant treatment in addition to surgery, including chemotherapy (n = 7), radiotherapy (n = 27), chemoradiation (n = 28). The primary tumor histopathological types were thymoma in 50 patients and thymic carcinoma in 33 patients. Masaoka staging information at the time of diagnosis were available in 63 patients (stage I–II, n = 23; III–IV, n = 40).

**Table 1:** Characteristics of patients.

Characteristic	Value
**Age, years**	
Median	52
Range	19–83
Gender, men/women, n	41/42
**Follow-up duration, months**	
Median	20
Range	2–118
**Histopathology, n (%)**	
Thymoma	50 (60.2)
AB type	9
B1 type	10
B2 type	14
B3 type	11
Unclassified	6
Thymic carcinoma	33 (39.8)
**Masaoka stage, n (%)**^**a**^	
Stage I–II	23 (36.5)
Stage III–IV	40 (63.5)
**Adjuvant treatment, n**	
Chemotherapy	7
Radiotherapy	27
Chemoradiation	28
Disease progression, n (%)	28 (33.7)
**PET/CT result, n (%)**	
Negative	33 (39.8)
Positive	50 (60.2)

^a^Available in 63 patients.

### FDG PET/CT performance

FDG PET/CT showed a positive finding in 50 patients and a negative finding in 33 patients. The findings of FDG PET/CT were histopathologically confirmed in 24 patients and were compared with clinical and/or imaging evaluation in the remaining 59. Forty patients with positive scans were confirmed with recurrent disease and were thus defined as truly positive. Ten patients were false positive, 33 patients were truly negative, and no patients in our series were false negative. The sensitivity, specificity, PPV, and NPV, and accuracy were 100%, 76.7%, 80%, 100%, and 87.9%, respectively. False-positive findings were related to post-surgical related uptake, and concomitant inflammatory disease affecting lymph nodes and lungs.

**Figure 1:**
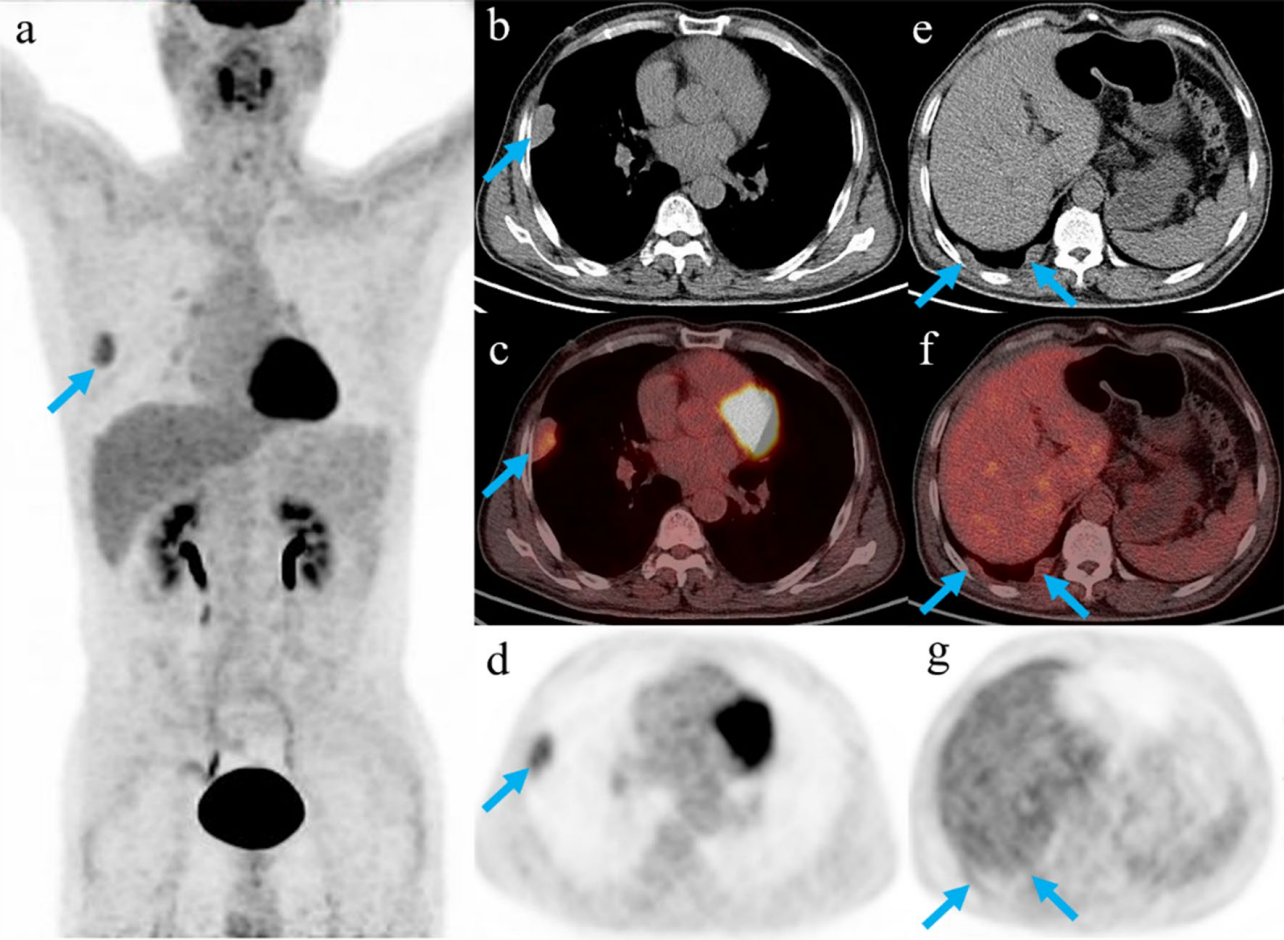
Representative images in a patient who was treated with surgery and radiotherapy for thymoma. Restaging FDG PET/CT showed increased uptake at the right pleura nodule (**a**–**d**, arrows; SUVmax, 4.3). In addition, several small-size nodules on the right pleura with negative FDG uptake were also observed on PET/CT (**e**–**g**, arrows). The pleural lesions were then surgically resected and confirmed by histopathology as recurrences of thymoma.

As described in Table 2, the most common mode of recurrence was regional recurrence, of which pleura was the most frequent site (Fig. 1). Of note was that the patients with distant sites of recurrence (liver, bone, lung) were all those with thymic carcinoma. Among the 40 patients with true positive FDG PET/CT findings, the SUVmax of the recurrent lesion with maximum uptake ranged from 2.4 to 27.5 (median, 7.0; mean, 7.95 ± 4.77). The lesion SUVmax in patients with thymic carcinoma (mean, 10.20 ± 5.77; range, 3.4‒27.5) was found to be significantly higher than that in patients with thymoma (mean 6.45 ± 3.32; range, 2.4‒15.5; *P* = 0.013).

**Table 2:** Sites of recurrence on FDG PET/CT.

Sites of recurrence	Frequency (n)^a^
**Regional recurrence**	36
Pleura	29
Pericardium	6
Lymph node	9
Local	5
Liver	6
Lung	1
Bone	2

^a^Some of the patients had more than one site of recurrence.

### FDG PET/CT and outcome

**Figure 2:**
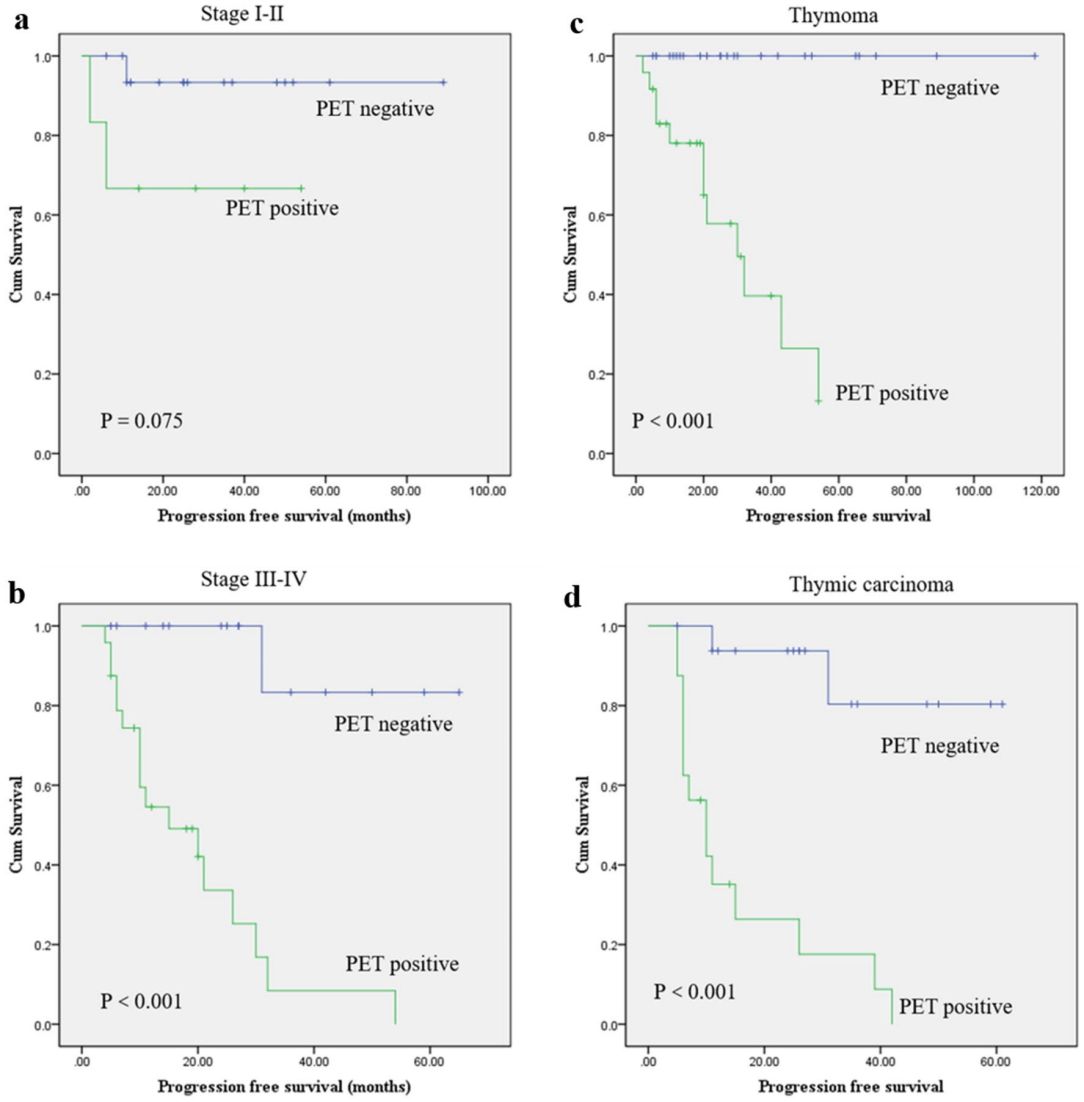
Kaplan Meier curves for progression-free survival (PFS) according to FDG PET/CT positivity for a different stage of Masaoka (**a**,**b**) and histopathology (**c**,**d**).

**Figure 3:**
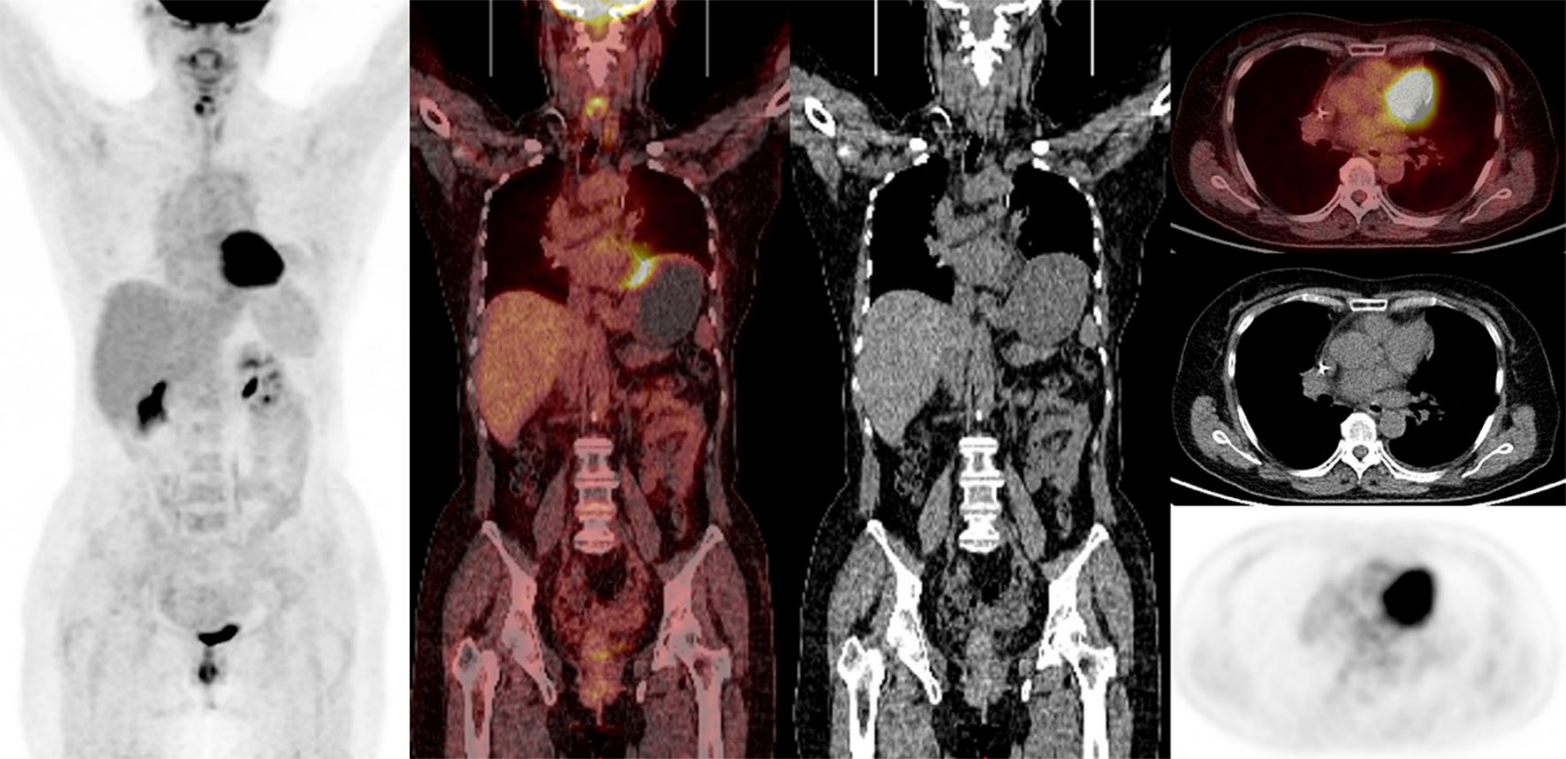
Representative images of an FDG PET/CT scan in a patient who was treated with surgery and adjuvant radiotherapy for stage III thymic carcinoma. Restaging PET/CT was negative. The patient remained disease-free during the follow-up period.

After a median follow-up period of 20 months, 28 patients had disease progression. Among the remaining 55 patients without disease progression, 12 had stable disease and 43 were disease-free. The 2-year and 3-year PFS rate was summarized in Table 3. By adding PET/CT-derived information (positive vs. negative) to Msaoka atage (I/II vs. III/IV) and histopathology (thymic carcinoma vs. thymoma), there was allowed stratification of the risk of disease progression (Fig. 2). Among 23 patients with Masaoka stages I and II, 6 patients were PET/CT positive, and patients with negative PET/CT scans had longer PFS than those with positive scans (P = 0.075; Fig. 2a). Among 40 patients with Masaoka stage III and IV, 24 patients were PET/CT positive. PET/CT negative patients had significantly longer PFS compared with those who were PET/CT negative (P < 0.001) (Fig. 2b; Fig. 3). FDG PET/CT also yielded added value if associated with histopathology. Among 50 patients with thymoma, 24 were PET/CT positive, and a negative PET/CT scan was significantly associated with better PFS (P < 0.001; Fig. 2c). Among 33 patients with thymic carcinoma, 26 had positive PET/CT findings. PET/CT negative patients had significantly better outcomes concerning PFS than PET/CT positive patients (P < 0.001; Fig. 2d).

**Table 3:** Kaplan–Meir analysis of 2-year and 3-year progression-free survival (PFS).

	2-year PFS (%)	3-year PFS (%)
**FDG PET/CT results (n = 83)**		
Positive	57	31
Negative	97	91
**Histopathology (n = 83)**		
Thymic carcinoma	61	50
Thymoma	79	70
**Masaoka staging (n = 63)**		
Stage I/II	86	86
Stage III/IV	60	36
**Histopathology and PET/CT**		
Positive PET/CT and TC	26	17
Negative PET/CT and TC	93	80
Positive PET/CT and thymoma	57	39
Negative PET/CT and thymoma	100	100
**Masaoka and PET/CT**		
Positive PET/CT and stage I–II	66	66
Negative PET/CT and stage I–II	93	93
Positive PET/CT and stage III–IV	33	8
Negative PET/CT and stage III–IV	100	83

We also investigated the prognostic value of SUVmax in those 40 patients with true positive results. ROC analysis revealed that the optimal SUVmax cut-off value was 5.3 (AUC = 0.732; sensitivity = 76.9%; specificity = 0.643) for estimating PFS. Kaplan–Meier survival curve revealed that patients with SUVmax > 5.3 tended to have shorter PFS than those with SUVmax ≤ 5.3, while the difference was not statistically significant (log-rank, P = 0.059).

## Discussion

Limited information is available on the clinical utility of FDG PET/CT for patients with recurrent TETs^20^. The present study demonstrated that FDG PET/CT had a high sensitivity of 100%, but a moderate specificity of 76.7% for detecting recurrence. Hatem et al. retrospectively evaluated 37 thymoma patients who underwent FDG PET/CT during postoperative follow-up and revealed a sensitivity of 82% and specificity of 95%^18^. Our study demonstrated a higher sensitivity compared to theirs, and this was probably due to the fact that our patient population also included those with thymic carcinoma, which generally had higher FDG activity than thymoma. In their study, the three false-negative results were all due to the small size of pleural nodules, measuring 1.3 cm, 2 cm, and 1.2 cm, respectively. As shown in Fig. 1, besides the hypermetabolic pleural lesion, the patient also had several relatively small lesions demonstrating negative uptake of FDG according to visual assessment. The recurrence of TETs on the pleura usually appears as multiple nodules. It might be difficult to determine the actual metabolic rate of those small nodules because of the partial volume effect, which might cause false-negative findings.

As with previous reports, the most common mode of recurrence was pleural recurrence. It is not clear whether pleural recurrences are related to particular biology of the tumor or whether they are the results of the seeding of the tumor^21^. We observed that all 6 patients in our series with distant site recurrences were those with thymic carcinoma. Similarly, Huang et al. also noted that recurrences in thymic carcinoma occurred significantly more frequently at distant sites than in thymoma^9^. Our analysis showed that the SUVmax of recurrent lesions from thymic carcinoma was significantly higher than that from thymoma. This finding supported the notion that these two thymic tumors are clinically distinct with thymic carcinoma showing more aggressive biomolecular behavior and higher metabolic rate.

FDG PET/CT in the restaging process has recently been demonstrated to play an important role in the prognostic assessment in other types of cancer. While it remains unclear whether restaging FDG PET/CT may also play a role in predicting prognosis in recurrent TETs. Previous studies have indicated the prognostic value of histopathology and Masaoka stage in TETs^6,9,22–25^. In this study, we evaluated the added prognostic value of FDG PET/CT to histopathology and Masaoka stage. The results demonstrated that in patients with the same Masaoka stage or histopathology, a positive FDG PET/CT scan had a worse outcome concerning PFS than those with a negative scan. Conversely, a negative PET/CT scan allows for the prediction of a more favorable outcome. Negative PET/CT scan might reflect a less aggressive biological nature of TETs, which is associated with better prognosis in terms of PFS. These results suggested that FDG PET/CT can further stratify patients with the same risk of progression and has a higher prognostic value in predicting PFS than Masaoka stage and histopathology alone.

Several limitations of this study should be pointed out. One limitation of the present study was related to the retrospective design. There is a lack of information regarding the presence or the absence of residual tumor after initial surgery, which could have affected the outcome. Second, the variable treatment regimens performed after the restaging scan may also have an impact on the outcome, while an additional analysis grouping patients based on the different treatment strategies was not feasible. Third, Masaoka staging data was not available for all patients as some of the patients received surgery at an outside hospital. The number of patients with stage I/II and positive PET/CT scan was relatively low, which might compromise the power of subgroup analysis. Additional limitation of this study was the small number of enrolled patients.

## Conclusions

FDG PET/CT showed a high diagnostic performance in patients with suspicion of recurrent TETs. In addition, FDG PET/CT showed an important prognostic value in assessing PFS.

## Methods

### Ethics approval and consent to participate

This study was approved by the Institutional Review Board of Peking Union Medical College Hospital. The requirement for informed consent was waived due to its retrospective nature and it was approved by the IRB of Peking Union Medical College Hospital. Investigations were carried out as per the rules of the Declaration of Helsinki of 1975, revised in 2013. All methods were performed in accordance with the institutional guidelines and regulations.

### Patients

Patients affected by TETs after definitive surgery who underwent FDG PET/CT for suspected recurrence between January 2010 and April 2020 were included in this retrospective study. The inclusion criteria were as follows: (1) thymoma or thymic carcinoma was diagnosed according to postoperative pathology; (2) FDG PET/CT was performed for suspicion of recurrence; (3) FDG PET/CT findings were confirmed by histology and/or conventional imaging (CT/MR) performed within 6 months; (4) availability of patients’ follow-up for at least 6 months after PET/CT scan; (5) the interval between surgery and FDG PET/CT scan over 4 weeks. A total of 83 patients met these criteria and were included in the final analysis. We performed per-patient analysis for the evaluation of FDG PET/CT performance (e.g., negative and positive scan). For the present study, we only analyzed the first post-treatment FDG PET/CT scan available for each patient; follow-up PET/CT scans were not evaluated for their diagnostic or prognostic value.

### Image analysis

All PET/CT images were retrospectively read by 2 experienced nuclear medicine physicians (GH and YJ), any disagreements were resolved by consensus. Since there is no reference standard value for maximum standard uptake value (SUVmax), we regard the PET/CT scan as positive based on the qualitative visual assessment. For example, PET/CT scan was defined as positive if the FDG activity in the lesion was moderately or markedly increased (moderate or strong uptake) relative to surrounding soft tissues. Conversely, lesions that showed no or weak FDG uptake (less than or equal to the surrounding soft tissue) were considered negative. SUVmax of the lesion with the highest metabolic activity in the patients with positive PET/CT scan was measured for statistical analysis. The final diagnosis was made by comparing the FDG PET/CT results with histopathology, if available, or with clinical data and/or imaging follow-up for at least 6 months. True positive PET/CT scan corresponded to an abnormal finding confirmed by histopathology or follow-up. PET/CT scan was deemed false positive if the histopathological results from a suspected FDG-positive lesion revealed no evidence of malignancy or the lesion remitted spontaneously without anticancer therapy during the follow-up period. A negative PET/CT scan was considered false negative if the lesion was detected by CT and was confirmed by histopathology or by clinical or imaging progression. True negative PET/CT scan corresponded to the absence of abnormal imaging findings confirmed by negative histopathological results or lack of recurrence during the follow-up. Recurrence patterns were recorded as local recurrence (disease occurring in the anterior mediastinum), regional recurrence (intrathoracic recurrence, including pleural/pericardial dissemination and mediastinal lymph node recurrence), and distant recurrence (intrapulmonary and extrathoracic recurrence)^26,27^.

### Statistical analysis

Continuous data are expressed as means and standard deviations. To evaluate the performance of FDG PET/CT in the restaging of TETs, we calculated the sensitivity, specificity, positive predictive value (PPV) and negative predictive value (NPV), and accuracy on a per-patient basis. Mann–Whitney U test was used to compare the variables between different groups. The survival curve of progression-free survival (PFS) was drawn by the Kaplan–Meier method, and differences in survival times between different groups were calculated using log-rank test. PFS was defined as the time from the PET/CT scan to the last follow-up or disease progression. The cutoff value of semi-quantitative parameters was determined by means of receiver-operating characteristic (ROC) analysis. P < 0.05 is considered to be statistically significant. The statistical analyses were performed using SPSS (IBM SPSS Statistics for Windows, Version 21.0. Armonk, NY).

## Data Availability

The data that support the findings of this study are available from the corresponding author, upon reasonable request.
